# Physical activity and health-related quality of life in chronic non-bacterial osteomyelitis

**DOI:** 10.1186/s12969-019-0351-4

**Published:** 2019-07-18

**Authors:** Nentwich Julia, Ruf Katharina, Girschick Hermann, Holl-Wieden Annette, Morbach Henner, Hebestreit Helge, Hofmann Christine

**Affiliations:** 1 Pediatric Rheumatology and Osteology, University Children’s Hospital Wuerzburg, Josef-Schneider-Str. 2, 97080 Wuerzburg, Germany; 2 Pediatric Pulmonology and Sports Medicine, University Children’s Hospital Wuerzburg, Wuerzburg, Germany; 3 Children’s Hospital, Vivantes Hospital im Friedrichshain, Berlin, Germany

**Keywords:** Chronic non-bacterial osteomyelitis, CRMO, HRQOL, Physical activity

## Abstract

**Background:**

Chronic non-bacterial osteomyelitis (CNO) is an autoinflammatory disorder of the skeletal system of yet unknown etiology. Patients present with local bone pain and inflammation and - to our experience - often suffer from functional impairment with significant disabilities of daily life. The objective of this study was to assess physical activity, fitness and health-related quality of life (HRQOL) in adolescents with established diagnosis of CNO versus healthy controls (HC).

**Methods:**

15 patients with CNO and 15 age and gender matched HC aged 13–18 years, completed questionnaires, performed an incremental exercise test with gas exchange measures up to voluntary fatigue and wore an accelerometer over 7 days at home to assess physical activity behavior.

**Results:**

At the time of assessment, 5 CNO patients were in clinical, one in radiological and 5 in clinical and radiological remission. 7 did not receive any therapy at the time of assessment. The results of the exercise test and of the accelerometry did not show any significant difference between CNO and HC. However, reported sports participation was lower in patients with CNO and PedsQL3.0 and 4.0 showed significant lower values in most of the scores indicating reduced HRQOL.

**Conclusion:**

Although most CNO patients showed a favorable course of disease without any relevant differences in objective measurements of physical activity and fitness versus HC at the time of assessment, questionnaires revealed perceived limitations. Further studies are needed to measure HRQOL and to validate questionnaires in patients with CNO against objective measures including more participants with a higher level of disease activity.

## Background

Chronic non-bacterial osteomyelitis (CNO) is an autoinflammatory disease of yet unknown etiology, which is mainly affecting the metaphysis of long bones, the pelvis, vertebral bodies and the clavicles or almost every site of the skeleton in children and adolescents [1–3]. Affected youths usually present with localized inflammation, including bone pain, swelling and warmth, often combined with functional impairment. Other associated symptoms including palmo-plantar pustulosis, psoriasis and inflammatory bowel disease have been reported [3–6]. Due to our experience, those symptoms are causing relevant limitations in motoric performance and age-appropriate development particularly in the chronically active disease course. Eventually this may result in a significant reduction of health-related quality of life (HRQoL) and physical activity.

The concept of HRQoL has been widely accepted in recent years as a patient-relevant outcome reflecting the impact of chronic illness in various health conditions [7]. HRQoL is a multidimensional concept that incorporates measures of physical symptoms, functional status, and disease impact on psychological and social functioning, in accordance with the definition of health proposed by the WHO. HRQoL instruments (generic and disease-specific) have been developed and used to measure the subjective impact of disease for different chronic diseases [7]. Severity and burden of disease manifestation in CNO cannot be correlated with and quantified by a laboratory value or imaging technique alone. And physical activity and physical function which have been directly linked to quality of life are often difficult to assess. Thus, there is an urgent need to identify comprehensive clinical measures, which assess the severity of disease manifestation in CNO to better understand the burden of this disease, to better monitor the course of disease in each individual patient and to evaluate the success of therapeutic strategies (e.g. physical training or pharmaceutical approaches). This pilot study addresses this need by assessing physical activity, fitness and HRQoL in a first group of patients with diagnosis of CNO versus age and gender matched healthy controls (HC) (age 13–18 years) using established measures including questionnaires, accelerometry and cycle ergometry [8–10]. We hypothesized that CNO patients have a significant reduction of HRQoL in questionnaires and objective measures. Primary outcome of the study was the assessment of HRQoL (PedsQL3.0 and 4.0). Secondary outcome was the assessment of objective measures including accelerometry and ergometry.

## Methods

The study was approved by the local ethics committee. 15 children (11 girls, 4 boys) diagnosed with CNO and 15 age and gender matched HC were recruited from the section of pediatric rheumatology and osteology at the University Children’s Hospital of Wuerzburg, Germany. At the time of assessment, all children were between 13 and 18 years of age (median 16 years).

Inclusion criteria for patients with CNO: diagnosis of CNO and age between 13 and 18 years. The diagnosis of CNO was based on the clinical picture, skeletal inflammatory MRI lesions, laboratory data and by exclusion of differential diagnoses. At the time of diagnosis, all patients with CNO had a multifocal disease and 8 had an initial diagnostic biopsy.

Each patient was compared to a healthy sex- (100% matching) and age- (CNO 16.0 years, HC 15.8 years, p 0.9593) matched volunteer (e.g. friend of the patient). Regarding their anthropometric data (height, body weight, calculated body surface area) HC did not differ significantly from patients with CNO. Great care was taken to recruit controls who were healthy and active, but did not participate in competitive sports at a higher than local level (anamnestic data). Participants (CNO and HC) who had competing diseases which might affect the physical activity were excluded from the study (exclusion criteria).

The participants came to the study center for a single visit. After informed consent was obtained from the guardians and verbal assent from the participants, questionnaires were completed, anthropometric measurements, clinical examination, blood analyses and an incremental exercise test on a cycle ergometer with gas exchange measures were performed. All participants were equipped with an accelerometer for 7 days following the clinic visit.

*Questionnaires*: The subjective physical activity was measured with an in-house established activity questionnaire, which combines the Habitual Activity Estimation Scale (HAES), 7-day Physical Recall Questionnaire (7D-PAR) and Lipid Research Clinics questionnaire (LRC), all translated into German and used before by Hebestreit et al. in juvenile idiopathic arthritis (JIA) and cystic fibrosis (Supplement). These questionnaires were combined with visual analogue scales (VAS) for pain at rest/during exercise, physical activity compared to other children, and fatigability after physical activity. For the assessment of quality of life and health perception the following questionnaires were administered: The DASS-G, Depression, Anxiety and Stress Scales – German version to assess depression, anxiety and stress. The Children Health Assessment Questionnaire (CHAQ), which relevance has been documented before in CNO by Beck et al. to record the amount of difficulty patients experience during daily life [4]. The German version of the Pediatric Quality of Life Inventory (PedsQL) was used to measure HRQoL. The PedsQL3.0 rheumatology module consists of five subscales and the PedsQL4.0 generic core scale consists of four subscales which can be combined into physical health and psychosocial health composite scales, as well as the PedsQL total score [11–13]. PedsQL scores range from 0 (being the worst) to 100 (being the best possible HRQoL). They have been used before in healthy children and children with JIA [13, 14].

*Cycle ergometry:* Aerobic fitness was assessed using the Godfrey protocol [15] and a calibrated commercial cycle ergometer (Ergoline, Ergoselect 100) with pulse oximetry (Nonin 7500, Nonin Medical, USA, Nellcor forehead sensor) and gas exchange measures (CPX/D, MedGraphics, St. Paul, MN, USA). Work rate was increased every minute by 10–20 W, depending on the subject’s height, up to voluntary fatigue. All results were compared to normal values depending on size and age of each patient, which were calculated as suggested by Godfrey et al. [16] for maximal power (Wmax), by Orenstein et al. [17] for maximal oxygen uptake (VO2max) and by Rowland [18] and Fairbarn et al. [19] for maximal heart rate (HFmax). All results were then stated in percent of the predicted value.

*Accelerometry:* All participants wore an accelerometer (GT3X, Actigraph) for 7 consecutive days on the hip to assess activity behavior objectively. Activity counts were averaged over a period of 5 s (epochs) and counts/ minute were calculated from these data. The measured counts were separated in different activity levels: sedentary, light (e.g. walking slowly, standing light work), moderate (e.g. walking very brisk, heavy cleaning) and vigorous (e.g. hiking, jogging) physical activity. A minimum recording time of 9 h on at least 2 week-days and 1 weekend-day was considered to yield valid data. Time spent in each activity level was determined separately for days during the week and the weekend. Due to the fact that there are no valid cut-offs for different activity levels in CNO, we chose to use cut-offs adjusted to the participant’s age as suggested by Troiano et al. [20] (Table 1).

**Table 1:** Cut-of-activity levels in counts per minute for accelerometry depending on participants´ age

	13y	14y	15y	16y	17y	>18y
Sedentary	0–99	0–99	0–99	0–99	0–99	0–99
Light	< 2393	< 2580	< 2781	< 3000	< 3239	< 2020
MPA	< 5375	< 5679	< 6007	< 6363	< 6751	< 5999
VPA	≥5375	≥5679	≥6007	≥6363	≥6751	≥5999

*MPA* time spent in moderate physical activity, *VPA* time spent in vigorous physical activity

For *statistical analysis* Wilcoxon sign rank tests were used to determine significant differences between groups of matched pairs (CNO and HC, case-control design)*.* Mann-Whitney-tests were used to determine significant differences between patients with CNO in complete remission and those who are not in complete remission. Spearman correlations between objective measures (accelerometry and cycle ergometry) and subjective measures (questionnaires) were calculated. Statistical significance was assumed at *p* < 0.05.

## Results

### Study population

Table 2 summarizes the clinical characteristics of all patients with CNO (medical history and at time of assessment). There was a median delay of 6.5 months in making the diagnosis after the first symptoms had appeared. In all patients, the disease onset was before 18 years of life (6 to 14 years, median 11 years). All patients had multifocal lesions at the time of diagnosis as shown on whole body MRI scan: in total, 107 radiologically defined inflammatory lesions were detected (median of 8 per patient). The head was involved in 2.0% of the 107 lesions, the extremities in 59.4% (upper extremities 12.9%, lower extremities 46.5%), the thorax in 7.9%, the spine in 14. 9%, and the pelvis in 15.8%. Most lesions of the extremities were localized in the metaphyses of the long bones close to the growth plate – only 1 lesion was in the diaphysis. 8 patients had an initial diagnostic biopsy for microbial and histological analyses. Comorbidities and other health problems are listed in Table 2. All patients started treatment with NSAID (after MRI/biopsy). Ten patients required additional treatment: azulfidine (9 patients), pamidronate (2 patients), MTX (1 patient), etanercept (1 patient), adalimumab (1 patient) and short term steroids (1 patient).

**Table 2:** Clinical characteristics of study patients with CNO

Patient	Sex	Medical history					At time of assessment			
		Age at diagnosis	Number of affected bones	Locations	Comorbidities/ problems	Treatment before assessment (in total)	Age	Treatment	CR	RR
1	F	12y	6	AC-joints, tibiae, femura	psoriasis	NSAID	14y	NSAID	no	no
2	M	14y	7	spine (Th4), femura, tibiae, humeri	acne	NSAID, Sulf	16y	NSAID, Sulf	yes	no
3	F	11y	8	tibia, femura, rib 5–7, spine (Th5–6)	scoliosis	NSAID, Sulf, Pam	14y	NSAID, Sulf	no	no
4	F	13y	6	rib 1 and 4, spine (Th4, Th6, Th7, Th11)	scoliosis	NSAID, ETA, Sulf, Pam	16y	NSAID, ETA	yes	no
5	F	9y	2	talus, tibia	–	NSAID	16y	none	yes	yes
6	F	11y	10	spine (L2, S1), acetabula, femura, tibiae, ossa metatarsalia III	M. Crohn, asthma	NSAID, Sulf	14y	NSAID, Sulf	yes	no
7	F	7y	10	spine (Th7), os sacrum, os pubis, acetabulum, femur, os metatarsale II, IV, calcaneus, tibiae, os cuboideum	OA	NSAID	16y	none	yes	yes
8	F	11y	5	tibiae, os sacrum, ossa ilea	scoliosis, asthma	NSAID, Sulf	14y	none	yes	yes
9	F	10y	9	spine (C6, Th2, Th8, L1), sternum, os sacrum, talus, ossa metatarsalia	FSGS	NSAID, Sulf	18y	none	yes	no
10	F	11y	4	tibia, ossa frontales, acetabulum	–	NSAID, Sulf	14y	NSAID, Sulf	no	no
11	F	12y	10	femur, acetabulum, rib 3 and 6, spine (Th4, Th9), humeri, calcaneus, ossa metatarsale II	arthritis	NSAID	16y	none	yes	no
12	M	12y	10	femura, ossa ilia, ossa ischiadica, ossa metatarsalia I, tibia, fibula	M. Crohn, asthma, atopic dermatitis	Ada, NSAID, MTX	15y	Ada	yes	yes
13	M	14y	2	os ilium, calcaneus	–	NSAID	15y	NSAID	no	no
14	F	8y	10	femura, talus, calcaneus, os naviculare, os metacarpale I, III, IV, os capitatum, os hamatum	Raynaud-syndrome, tendovaginitis	NSAID, Sulf	17y	none	no	yes
15	M	8y	8	femura, os pubis, ossa metatarsalia, calcanei	obesity	NSAID, Sulf	17y	none	yes	yes

The medical history is depicted in the first part, the clinical characteristics at time of study assessment in the last four columns

*F* female, *M* male, *y* years, *C* cervical, *L* lumbar, *Th* thoracic, *S* sacral, *AC* acromioclavicular, *FSGS* focal segmental glomerulosclerosis, *OA* oligoarthritis, *asthma* asthma bronchiale, *NSAID* non-steroidal anti-inflammatory drugs, *Sulf* sulfasalazine, *Pam* pamidronate, *ETA* etanercept, *Ada* adalimumab, *MTX* methotrexate, *CR* clinical remission, *RR* radiological remission

### Clinical features at the time of assessment

At the time of assessment (mean age 16.0 years, mean time after making the diagnosis/starting treatment 5.7 years) 33% (5/15) were in clinical remission (CR, no clinical symptoms) and radiological remission (RR, no inflammatory MRI lesions) with a mean time of 20 months (min. 3, max. 72 months. 33% (5/15) were in CR but not RR with a mean time of12 months (min. 2, max. 24 months). 7% (1/15) were in RR but not CR and 27% (4/15) were neither in CR nor in RR.

At the time of assessment 47% (7/15) did not receive any therapy, 20% (3/15) were treated only with naproxen, 20% (3/15) with naproxen and sulfasalazine, 6% (1/15) with etanercept and naproxen and 6% (1/15) with adalimumab.

Gender and age matched HC (mean age 15.8 years) did not show significant differences in length and body weight Z-scores and in BMI. 2 HC were diagnosed with mild asthma, which did not require any medical treatment, 1 with Factor-V-deficiency and none received any medical therapy. Results of the laboratory parameters (CRP, ESR and leucocytes) did not reveal significant differences between patients with CNO and HC.

**Fig. 1:**
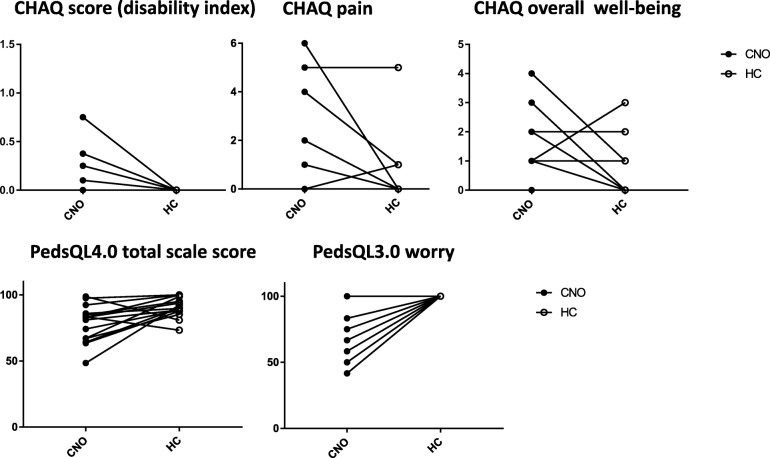
CHAQ-score (disability index), CHAQ pain and CHAQ overall well-being (**a**), PedsQL4.0 total scale score and PedsQL4.0 worry (**b**) in 15 patients with CNO and 15 sex – and age-matched HC. Selected items in individual patients and their controls (case-control design) that are of special interest and discussed in the manuscript (see Table 4)

Results of the exercise test (cycle ergometry) and the accelerometry (% of time spent in each activity level) are summarized in Table 3. There were no significant differences between patients with CNO and HC in all parameters of both assessments *(also* Fig. 1*).*

**Table 3:** Results of exercise testing and accelerometry: CNO compared to HC

	CNO (*n* = 15)	HC (n = 15)	*P*-values
Cycle ergometry			
Wpeak (% predicted)	106 [90; 114]	99 [94; 107]	0.8040
HRpeak (% predicted)	95 [93; 100]	98 [93; 101]	0.6387
VO2peak (% predicted)	95 [87; 109]	93 [86; 100]	0.7197
RQmax	1.14 [1.08; 1.17]	1.17 [1.14; 1.19]	0.0488*
Accelerometry (% of time spent in each activity level)			
sedentary physical activity	66 [62; 72]	69 [60; 78]	0.6257
light physical activity	25 [21; 27]	23 [18; 29]	0.8077
moderate physical activity	7 [7; 8]	7 [5; 10]	0.9515
vigorous physical activity	1 [0; 2]	1 [1; 2]	0.6587

Results presented as median [95% confidence interval of median]. Statistical analysis performed using Wilcoxon sign rank test. Results of the exercise test (cycle ergometry) and of the accelerometry (measured for 7 consecutive days at the right hip) did not show any significant differences between patients with CNO and HC

Wpeak: maximal power in % predicted according to Godfrey-protocol [16], HRpeak: peak heart rate in % predicted according to Rowland for teenagers between 13 and 17 years [18] and Fairbarn [19] for adults. VO2peak: peak oxygen update in %predicted according to Orenstein [17], RQmax: peak respiratory quotient

Table 4 summarizes the results of the questionnaires (CHAQ, PedsQL3.0 and 4.0, and LRC). The CHAQ-score disability index did not show any difference between patients with CNO and HC. The pain subscale of the CHAQ appeared slightly higher in the patients without being significantly different from HC and with an average of 1 on a VAS 0–10 very low. Overall well-being was reported significantly worse by the patients with CNO compared to the HC. The PedsQL3.0 revealed significantly lower scores in patients with CNO in three subscales: “pain and hurt”, “daily activities” and “communication” indicating more problems in these areas. Interestingly a highly significant difference was found in the category “worry” *(*Table 4 *and* Fig. 1*).* The PedsQL4.0 also showed significantly lower scores in the categories “physical functioning” and “school functioning” as well as in the total scale score in patients with CNO *(also* Fig. 1*).* Significant differences were also observed in the activity questionnaire. In particular, reported sports participation was significantly lower in patients with CNO (2[0;2] vs. 2[2;2], 0: no participation, 1: limited participation, 2: unlimited participation, p 0.0313). 5 patients with CNO did not participate in any sport and one did participate with restrictions. Pain during exercise and limitation of physical activity was significantly more frequent in patients with CNO compared with HC as reported in the activity questionnaires with VAS. No further significant differences could be found in the activity questionnaire with VAS and the DASS-G questionnaire (data not shown).

**Table 4:** Results of questionnaires: CHAQ, PedsQL3.0 and 4.0, activity questionnaire

	CNO (n=15)	HC (n=15)	*P*-values
CHAQ			
CHAQ-score (disability index)	0 [0;0]	0 [0; 0]	0.1250
pain (VAS 0-10)	1 [0; 2]	0 [0; 0]	0.1328
overall well-being (VAS 0-10)	2 [1; 3]	0 [0; 1]	**0.0107**
PedsQL3.0 rheumatology module			
pain and hurt	81 [69; 94]	94 [81; 100]	**0.0458**
daily activities	100 [95; 100]	100 [100; 100]	**0.0625**
treatment	93 [79; 100]	93 [82; 100]	0.8696
worry	75 [58; 100]	100 [100; 100]	0.0010***
communication	92 [67; 100]	100 [92; 100]	0.03*
PedsQL4.0 generic core scale			
total scale score	83 [67; 86]	90 [88; 99]	0.02*
physical functioning	78 [69; 94]	94 [91; 100]	0.01**
emotional functioning	75 [55; 95]	85 [75; 95]	0.11
social functioning	95 [85; 100]	100 [90; 100]	0.18
school functioning	75 [55; 85]	85 [80; 100]	0.03*
Activity questionnaire with VAS (LRC)			
LRC_original	2 [1; 2]	1 [0; 1]	0.2500
LRC_2point	1 [0; 1]	0 [0; 0]	0.69
LRC_4point	2 [0; 2]	0 [0; 1]	0.2500

Results presented as median [95% confidence interval of median]. Statistical analysis performed using Wilcoxon sign rank test

Lipid Research Clinics (LRC): LRC original with activity category 0 (inactive), 1 (moderate active) and 2 (active); LRC 2 point with activity category 0 (inactive) and 1 (active); LRC 4 point with activity category 0 (very low activity), 1 (low activity), 2 (moderate activity) and 3 (high activity) [8, 10]

CHAQ with CHAQ score (disability index) 0–3 and pain and overall well-being on 10 point VAS. PedsQL 3.0 and 4.0 Scale scores with different subscale scores (100 possible points), higher scores indicate better HRQoL

For the routine assessment of patients´ physical activity in a clinical setting, a quick and easily accessible tool at low costs is required; therefore, questionnaires might be best suited. Therefore we correlated the different items assessed in the questionnaires with objective parameters assessed with accelerometry and cycle ergometry. Table 5 summarizes the relationship between measurable physical activity and self-reported mood and fitness through questionnaires (PedsQL3.0 and activity questionnaire with VAS) in patients with CNO. Interestingly only some correlations could be found. Patients still complaining about pain seem to be less active and have less physical fitness. Significant correlations were observed between the category “pain and hurt” of the PedsQL 3.0 and time spent in VPA, as well as the HRpeak. The category “treatment” (PedsQL 3.0) correlated with VO_2_peak. Furthermore some VAS of the activity questionnaire (frequency/intensity of pain at rest, frequency/intensity of pain during exercise) correlated significantly with time spent in VPA and MVPA as well as with peak heart rate during the exercise test. There were no correlations between scales from questionnaires and objective measures of physical activity or fitness (accelerometry and cycle ergometry) not depicted in Table 5.

**Table 5:** Correlations relating objectively measured physical activity (results of the exercise test and accelerometry) with subjective physical activity assessed by questionnaires

	MPA	*p*	VPA	*p*	MVPA	*p*	W peak	*p*	HR peak	*p*	VO2 peak	*p*	RQ peak	*p*
	r		r		r		r		r		r		r	
PedsQL3.0														
pain and hurt	0.44	0.1	0.62	0.0166*	0.45	0.09	0.09	0.74	0.64	0.0097**	0.2	0.46	− 0.13	0.65
daily activities	−0.08	0.77	0.23	0.4	− 0.05	0.85	−0.05	0.85	0.42	0.12	−0.1	0.73	0.06	0.84
treatment	0.24	0.39	0.13	0.64	0.11	0.7	0.4	0.14	0.18	0.51	0.58	0.0267*	−0.09	0.75
worry	−0.26	0.34	−0.15	0.58	−0.32	0.24	0.35	0.2	−0.13	0.65	0.12	0.66	0	0.99
communication	−0.35	0.19	−0.02	0.96	−0.36	0.18	0.13	0.65	0.33	0.23	0.15	0.59	0.04	0.89
Activity questionnaire (with VAS)														
pain at rest - frequency	−0.16	0.56	−0.41	0.13	−0.24	0.38	0.24	0.39	−0.59	0.0219*	0.16	0.56	0.18	0.53
pain at rest - intensity	−0.53	0.09	−0.7	0.0212*	−0.7	0.0212*	0.38	0.25	−0.56	0.08	0.17	0.63	0.05	0.88
pain during exercise- frequency	−0.2	0.47	−0.6	0.0209*	−0.24	0.39	0.04	0.89	−0.57	0.0277*	−0.11	0.68	−0.25	0.37
pain during exercise- intensity	−0.48	0.12	−0.76	0.0046**	−0.55	0.07	0.35	0.26	−0.56	0.06	0.15	0.65	−0.42	0.17
limitation of physical activity compared with other children	−0.06	0.84	−0.13	0.64	−0.03	0.92	−0.12	0.66	−0.09	0.75	−0.07	0.79	0.32	0.24
fatigability after physical activity	−0.46	0.09	−0.3	0.27	−0.42	0.12	0.03	0.92	−0.37	0.17	−0.09	0.76	0.16	0.56

Results are Spearman correlation coefficients (r) and the respective probability of a type I error (p)

*MPA* time spent in moderate physical activity, *VPA* time spent in vigorous physical activity, *MVPA* time spent in moderate and vigorous physical activity, *Wpeak* maximal power in cycle ergometry, *HRpeak* peak heart rate in cycle ergometry, *VO2peak* peak oxygen uptake in cycle ergometry, *RQpeak* peak respiratory quotient, *VAS* visual analogue scale

Explorative analyses of the data from the 15 patients with CNO indicated statistically significant differences in the LRC score as well as in the VAS of the CHAQ between patients in complete remission (CR and RR, *n* = 4) and those who were not in complete remission (*n* = 11). Interestingly the VAS for pain (0[0;2] vs. 3[0;6], p 0.0455) and overall well-being (1[0;2] vs. 3[2;4], p 0.0278) was significantly lower in patients who were in complete remission; the CHAQ score (disability index) did not show any differences (0[0;0.25] vs. 0[0;0.75], p 0.8791). Furthermore, patients in complete remission spent more time in sports (4.5[1.5;6] vs. 0.25[0;2] hours, p 0.0183) and had higher scores in the LRC of the activity questionnaire (LRC_orginal 2[1;2] vs. 0.5[0;1], p 0.0161, LRC_2point 1[0;1] vs. 0[0;0], p 0.0256, LRC_4point 2[0;2] vs. 0[0;1], p 0.0183) There were no significant effects of disease status – remission or no remission - on other scales from questionnaires, physical activity or fitness within the CNO group.

## Discussion

The aim of this study was to measure physical activity and HRQoL of patients with CNO in comparison to age and gender matched healthy controls. There is a definite lack of knowledge in the literature about these items. Although most of our patients with a more than 5 year history of CNO showed a favorable course of disease without any relevant differences in objective measurements of physical activity and fitness versus HC at the time of assessment, self-reported measures of physical activity and quality of life revealed limitations.

### Self-reported measurements of physical activity and HRQoL

The **PedsQL3.0** revealed significantly lower scores in patients with CNO and interestingly high significance was found in the category “worry” **(**Fig. 1**)**. The **PedsQL4.0** showed significantly lower scores in the categories “physical functioning” and “school functioning” as well as in the total scale score. The PedsQL generic core scales and rheumatology module have been shown to be reliable, valid, sensitive to disease severity, and responsive to change in JIA but to our knowledge have not been used in CNO [13]. Interestingly in our study data for PedsQL3.0 and 4.0 seem somehow contradictory. PedsQL3.0 revealed a high significant difference in the category “worry” whereas no significance could be found in the category “emotional functioning” in the PedsQL4.0. In the PedsQL4.0 the physical domain seems to be more important **(**Table 4**,** Fig. 1**)**.

According to Varni et al. the PedsQL minimally clinical important difference (MCID) is 4.4 points and an optimal HRQoL is defined as a score no less than one MCID below the mean of healthy children on the PedsQL total scale score [12]. The mean score of healthy children in our study is 90 (according to Varni et al. 83) and a PedsQL total scale score of below 85.6 (according to Varni et al. 78.6) is defined as sub-optimal [12]. Thus, in our study CNO patients achieve an suboptimal HRQoL with a PedsQL total scale score of 83, but compared to other studies the reduction seems to be very moderate [12, 21]. 6 of our CNO patients (40%) have a PedsQL 4.0 total scale score of below 75 after a more than 5 year history of CNO. 2 of these patients were in complete remission, one in clinical, one in radiological and 2 neither in clinical nor radiological remission.

The **CHAQ** score which is the most widely utilized functional status measure in pediatric rheumatology and shown to be valid, reliable, and sensitive in JIA was 0 in CNO patients. This is in accordance to our previous findings in patients treated for 12 months (Beck et al.) or longer (not published). Mean treatment period in our cohort was more than 5 years and one third of the patients was in complete and one in clinical but not radiological remission. When compared with JIA, however, the CHAQ score at the time of diagnosis in CNO is known to show much lower levels [4]. The CHAQ overall well-being on a VAS was significantly worse (0 vs. 2) but with a mean of 2 still moderate compared to HC.

In the **LRC** in particular reported sports participation was significantly lower in patients with CNO. These data suggest that some patients have an impairment of their HRQoL long after inflammatory lesions have ceased, which is likely attributable to neuropsychological factors and probably aspects of persisting pain although pain at the time of assessment was rated low in the CHAQ (CHAQ pain 1 on a VAS). But the category “pain and hurt” of the PedsQL3.0 rheumatology module showed significantly more pain in CNO patients. Given the limitation of a very small number of patients with CNO and the low inflammatory activity level in the pilot study it could not be clarified if “pain” and “limitations” were really physical or rather emotional problems.

Recently Silier et al. published the results of a patient survey which revealed the negative impact of CNO on daily life including family relationships, friendships and work/school and highlighted the need for better psychosocial support such as guidance counselling or psychological support [22].

Participation in physical exercise is encouraged especially in “anxious” patients with CNO in radiological or complete remission. Beside cardiovascular benefits physical exercise may prevent loss of bone mineral density associated with chronic inflammatory conditions [23]. In CNO loss of BMD has not been described so far as most of the metaphyseal lesions are osteoblastic and spine lesions osteo-destructive [24].

### HRQoL in other diseases

Similar to our data in CNO, several studies have shown that newly diagnosed patients with JIA have a lower HRQoL and they gradually improve over time resulting in an approximation of the patients´ PedsQL values to those of healthy peers after 3 years of rheumatologic care [11, 21]. According to Listing et al. only a quarter of the patients with JIA had a suboptimal HRQoL after 3 years of treatment [21]. Chronic pain, persistence of high levels of disease activity, familial burden and functional impairment have been identified as indicators of a suboptimal HRQoL over the course of disease in JIA [14, 21, 25]. However, to our knowledge it has remained unclear why patients with clinically inactive JIA still report a reduced quality of life [14].

Similar findings have been reported by Hebestreit et al. for HLA-B27 positive juvenile spondylarthropathy in whom disease is inactive or in remission [8]. The authors observed a lower aerobic fitness with a tendency for lower Wpeak and VO2peak during a continuous incremental cycling task versus healthy controls. The unfavorable long-term effects of unfounded fear on exercise as a possible indicator for a flare of disease have also been shown for the first time in schoolchildren whose parents thought something was wrong with their child’s heart [26].

### Pilot model project in a rare disease

Nevertheless, it is important to identify possible predictors of a suboptimal quality of life like family burden, pain and functional impairment as well as clinical and radiological disease activity in the course of CNO in clinical practice to be able to address needs at an early stage of the disease with targeted treatment strategies and support measures of the families (e.g. psychological support, individual established sports and exercise counselling).

The strength of the study is the objective measure of aerobic fitness with a continuous incremental cycling task which to our knowledge has never been published in patients with CNO before. However, accelerometry and cycle ergometry are expensive and time consuming and in particular the latter requires specialized equipment. For the assessment of patients´ physical activity in routine clinical care questionnaires, quick and easy accessible and cheap, seem to be best suited. Interestingly in our study only some correlations between objective measures of physical activity behavior or aerobic fitness and scales from used questionnaires could be found. Further on only self-reported HRQoL questionnaires have been used in our cohort. Parents of sick patients (different diseases) describe on average a lower HRQoL than children themselves [27], resulting in an underestimation in HRQoL.

However, this is a pilot project with a very small number of patients with CNO, a rare autoinflammatory bone disease. Our CNO patients have a more than 5 year history of CNO with most of them being in clinical and/or radiological remission at the time of assessment. We did not present data on aerobic fitness and HRQoL at time of diagnosis or longitudinal data at different time points during the course of disease.

## Conclusion

In conclusion, our small study shows that patients with CNO (even in clinical and/or radiological remission) may have an impairment of their HRQoL with lower scores in different questionnaires. Objective measurements of physical activity and fitness revealed no differences versus healthy controls in our study. These are crucial findings for the counselling of patients and their parents. Psychological factors like unfounded fear or worry on exercise seem to be important determinants of fitness in our patients highlighting the need for better psychosocial support during treatment. Patients should be encouraged to exercise as soon as inflammation ceases and as much as medically acceptable followed by an individual sports and exercise counselling.

Further studies are needed to measure HRQoL and to validate questionnaires in patients with CNO against objective measures including more participants with a higher level of disease activity. This observation in a small group of patients with CNO may serve as a model project for other rare pediatric diseases which result in a relevant movement disorder.

## Data Availability

The datasets used and/or analysed during the current study are available from the corresponding author on reasonable request.

## Abbreviations

7D-PAR: 7-day Physical Recall Questionnaire
BMD: Bone mineral density
CHAQ: Children Health Assessment Questionnaire
CNO: Chronic non-bacterial osteomyelitis
HAES: Habitual Activity Estimation Scale
HC: Healthy controls
HRQoL: Health-related quality of life
JIA: Juvenile idiopathic arthritis and
LRC: Lipid Research Clinics questionnaire
MPA: Moderate physical activity
MVPA: Moderate and vigorous activity
PedsQL: Pediatric Quality of Life Inventory
VAS: Visual analogue scale
VPA: vigorous physical activity
