# Correction to: Physical activity and health-related quality of life in chronic non-bacterial osteomyelitis

**DOI:** 10.1186/s12969-019-0394-6

**Published:** 2020-02-03

**Authors:** Julia Nentwich, Katharina Ruf, Hermann Girschick, Annette Holl-Wieden, Henner Morbach, Helge Hebestreit, Christine Hofmann

**Affiliations:** 1 Pediatric Rheumatology and Osteology, University Children’s Hospital Wuerzburg, Josef-Schneider-Str. 2, 97080 Wuerzburg, Germany; 2 Pediatric Pulmonology and Sports Medicine, University Children’s Hospital Wuerzburg, Wuerzburg, Germany; 3 Children’s Hospital, Vivantes Hospital im Friedrichshain, Berlin, Germany

**Correction to: Pediatr Rheumatol Online J (2019) 17:45**

**https://doi.org/10.1186/s12969-019-0351-4**

Following publication of the original article [1], we have been notified that the authors’ first names and last names are presented in wrong order. The presentation of names, thus, should be as follows:

Julia Nentwich 1, Katharina Ruf 2, Hermann Girschick3, Annette Holl-Wieden 1, Henner Morbach 1, Helge Hebestreit 2 and Christine Hofmann 1*
